# Effects of synthetic cohesin-containing scaffold protein architecture on binding dockerin-enzyme fusions on the surface of *Lactococcus lactis*

**DOI:** 10.1186/1475-2859-11-160

**Published:** 2012-12-15

**Authors:** Andrew S Wieczorek, Vincent JJ Martin

**Affiliations:** 1 Department of Biology, Centre for Structural and Functional Genomics, Concordia University, Montréal, Québec, H4B 1R6, Canada

## Abstract

**Background:**

The microbial synthesis of fuels, commodity chemicals, and bioactive compounds necessitates the assemblage of multiple enzyme activities to carry out sequential chemical reactions, often via substrate channeling by means of multi-domain or multi-enzyme complexes. Engineering the controlled incorporation of enzymes in recombinant protein complexes is therefore of interest. The cellulosome of *Clostridium thermocellum* is an extracellular enzyme complex that efficiently hydrolyzes crystalline cellulose. Enzymes interact with protein scaffolds via type 1 dockerin/cohesin interactions, while scaffolds in turn bind surface anchor proteins by means of type 2 dockerin/cohesin interactions, which demonstrate a different binding specificity than their type 1 counterparts. Recombinant chimeric scaffold proteins containing cohesins of different specificity allow binding of multiple enzymes to specific sites within an engineered complex.

**Results:**

We report the successful display of engineered chimeric scaffold proteins containing both type 1 and type 2 cohesins on the surface of *Lactococcus lactis* cells. The chimeric scaffold proteins were able to form complexes with the *Escherichia coli* β-glucuronidase fused to either type 1 or type 2 dockerin, and differences in binding efficiencies were correlated with scaffold architecture. We used *E. coli* β-galactosidase, also fused to type 1 or type 2 dockerins, to demonstrate the targeted incorporation of two enzymes into the complexes. The simultaneous binding of enzyme pairs each containing a different dockerin resulted in bi-enzymatic complexes tethered to the cell surface. The sequential binding of the two enzymes yielded insights into parameters affecting assembly of the complex such as protein size and position within the scaffold.

**Conclusions:**

The spatial organization of enzymes into complexes is an important strategy for increasing the efficiency of biochemical pathways. In this study, chimeric protein scaffolds consisting of type 1 and type 2 cohesins anchored on the surface of *L. lactis* allowed for the controlled positioning of dockerin-fused reporter enzymes onto the scaffolds. By binding single enzymes or enzyme pairs to the scaffolds, our data also suggest that the size and relative positions of enzymes can affect the catalytic profiles of the resulting complexes. These insights will be of great value as we engineer more advanced scaffold-guided protein complexes to optimize biochemical pathways.

## Background

The spatial organization of enzymes through compartmentalization in organelles, co-localization on membranes or assembly in complexes using protein scaffolds or fusions plays an important role in controlling the flow of metabolites in a cell [1,2]. Spatially organized multi-enzyme pathways can serve many functions such as substrate channeling to reduce the loss of intermediates to competing side reactions. Channeling can also be used to prevent the accumulation of toxic or unstable metabolites [3]. Higher localized concentration of proteins and metabolites, dubbed molecular crowding, also decreases product/reactant diffusion and increases yields and rates of metabolite production [1]. Spatial organization is also used to control the stoichiometry of the proteins that make up the complex and to protect proteins from degradation [2]. Synergism between enzymes in a complex can also result in an activity that is higher than the sum of its parts, as demonstrated by cellulosomes [4]. Inspired by nature and driven by the need to achieve high production yields in industrial microbes, metabolic engineers have started tinkering with the spatial organization of enzymes in cells using synthetic protein scaffolds and organelles [5-7].

Cellulosomes have been a significant source of inspiration for the engineering of extracellular protein scaffolds [8-11]. Cellulosomes are protein complexes comprised of a multitude of hydrolytic enzymes with varying catalytic properties that associate with a central scaffold protein to enhance synergy when degrading cellulose [12,13]. In *Clostridium thermocellum,* the scaffold protein CipA is anchored to the cell surface via anchor proteins such as OlpB and SdbA [14,15], yielding an extra level of synergy resulting from cellulose-enzyme-microbe (CEM) ternary complexes [16-23]. The assembly of the protein complex is mediated via interactions of non-catalytic dockerin and cohesin domains, where type 1 and type 2 domains exhibit distinctive binding specificities, as do dockerin and cohesin partners from different species [4]. Cellulosomal enzymes carry type 1 dockerin (dock1) domains and interact with any of the nine type 1 cohesin (coh1) domains found on CipA [13], while CipA itself has a type 2 dockerin (dock2), which interacts with type 2 cohesins (coh2) on anchor proteins OlpB and SdbA [14,15].

Recombinant mini-cellulosomes have been assembled *in vitro* from individual components produced separately in *Escherichia coli* and *Bacillus subtilis*[8,9,24-26]. The *in vivo* assembly of similar complexes has also been achieved in hosts such as *Clostridium acetobutylicum* and *B. subtilis* where proteins were targeted for secretion into the supernatant [10,27,28]*.* The successful anchoring of functional mini-cellulosomes on the surface of *Saccharomyces cerevisiae* has been described as well, for the purpose of converting cellulose to ethanol [11,29-31]. In these studies, chimeric scaffolds were engineered by combining type 1 cohesins from different bacterial species, or with other non-cohesin ligand-binding domains.

**Figure 1:**
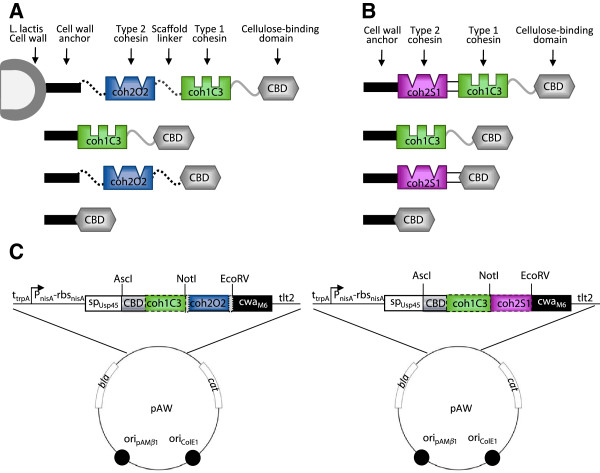
**Depiction of chimeric scaffold proteins and expression cassettes. **(**A**) Chimeric protein scaffolds generated as fusions of the CipA type 1 cohesin coh1C3 (green) with the OlpB type 2 cohesin coh2C2 (blue) and cellulose binding domain CBD (grey). Linkers between cohesin domains, the cell anchor, or the CBD are derived from OlpB (black dotted) or CipA (grey). Optional linkers are represented by dotted lines. (**B**) Chimeric protein scaffolds generated as fusions of CipA type 1 cohesin coh1C3 (green) with the type 2 cohesin of SdbA (purple) and cellulose binding domain CBD (grey). Double lines represent direct fusion of two domains without a linker sequence. (**C**) Scaffold expression cassettes showing the N-terminal signal peptide from the lactococcal Usp45 secreted protein (sp_Usp45_) and the cell wall anchor motif of the M6 protein (cwa_M6_). Expression of the cassettes is under the control of the *nisA* nisin-inducible promoter (*P*_*nisA*_) and ribosome-binding site (*rbs*_*nisA*_) from *L. lactis*. The transcriptional terminators of the *rrnB* operon (*tlt2)* and *trpA* gene (*t*_*trpA*_) are located upstream and downstream of the expression cassette, respectively. Optional DNA sequences encoding certain modules are surrounded by dotted lines.

The lactic acid bacterium *Lactococcus lactis* is an established host for the production of lactic acid [32], bioactive compounds [33], enzymes [34-36], interleukins [37] and as a live vaccine for the delivery of antigens [38-41]. We recently reported on the successful display of scaffold proteins containing only type 1 cohesins on the surface of *L. lactis*[42]. We have since expanded on this work, and in the present study report on engineered strains capable of displaying chimeric scaffold proteins resulting from the fusion of cohesin(s) of the CipA protein with a cohesin from OlpB or SdbA anchor proteins (Figure 1). The most complex scaffolds contained type 1 and type 2 cohesins, as well as a cellulose-binding domain (CBD), and were composed solely of building blocks of the *C. thermocellum* cellulosome. The effects of protein scaffold architecture were investigated by using or excluding linker sequences between cohesins, by varying the number and origin of cohesins in the chimeric scaffold, and by changing the order in which enzymes were localized within the complex. The specificity and efficiency at binding the dockerin containing *E. coli* β-glucuronidase (UidA) and β-galactosidase (LacZ) reporter enzymes to each of the synthetic scaffolds was tested.

## Results

### UidA-dock1 binds to coh2O2-coh1C3 chimeric proteins displayed on *L. Lactis*

**Figure 2:**
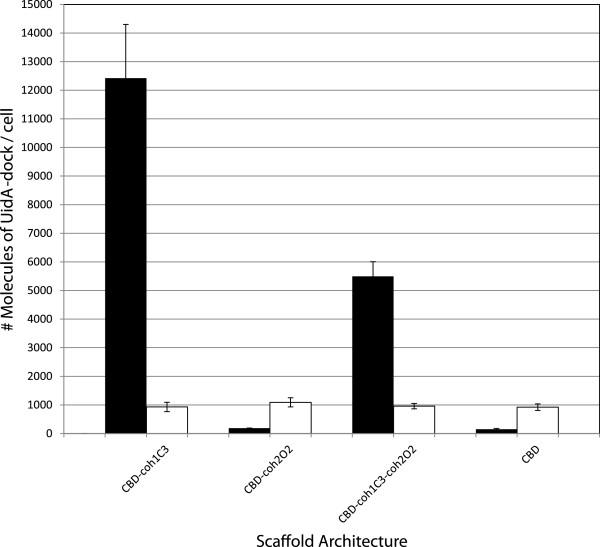
***In vivo *binding of UidA-dock1 and UidA-dock2 on *L. lactis *cells displaying coh1C3-coh2C2 chimeric scaffold proteins.** Cells displaying chimeric scaffolds were tested for their ability to bind UidA-dock1 or UidA-dock2. Scaffolds were comprised of a type 1 cohesin domain, a type 2 cohesin domain, both a type 1 and type 2 cohesin domain, or no cohesin domain. Quantification of enzymes was carried out using the calculated specific activity of purified enzyme, and the known amount of cells in each sample. The number of molecules bound to *L. lactis* cells corresponds to equivalent amounts of functional cohesin assuming a theoretical 1:1 ratio of dockerin to cohesin binding. Bars represent the number of UidA-dock1 molecules (black bars) and UidA-dock2 molecules (white bars) successfully associated with the scaffolds.

Chimeric scaffold proteins containing cohesins of different specificity were expressed as fusions with the N-terminal signal peptide from the lactococcal Usp45 secreted protein (sp_Usp45_) [43] and under control of the *nisA* nisin-inducible promoter (*P*_*nisA*_) and ribosome-binding site (*rbs*_*nisA*_) from *L. lactis*[44,45]. For simplicity of scaffold nomenclature, the number preceding the uppercase letter represents the type of cohesin (type 1=coh1 and type 2=coh2), the uppercase letter represents the protein of origin (CipA=C, OlpB=O and SdbA=S) and the number proceeding the uppercase letter represents the relative position of the cohesin from the N-terminus of the protein of origin. The first chimeric protein scaffold architecture tested in *L. lactis* consisted of the cellulose binding domain (CBD), the third type 1 cohesin of CipA (coh1C3), as well as the second type 2 cohesin domain of OlpB (coh2O2). Our previous work suggested the possibility that the CBD may aid in the secretion of larger scaffolds, it was therefore included in all coh2O2 fusions [42]. In order to investigate the effects of including linkers between scaffold domains on the efficiency of enzyme binding, the chimeric proteins were constructed with and without linker sequences between the two cohesins and between the cohesin and the cell wall anchor domain (Figure 1A). *In vivo* binding assays were used to show binding of the dockerin-containing β-glucuronidase (UidA-dock1) to the scaffold and to verify the specificity of dockerin 1 (dock1) to coh1C3 interaction. The dock1 domain used was derived from cellulosomal enzyme CelS, which is capable of binding any of the nine type 1 cohesins of CipA [46]. Cells displaying chimeric scaffolds containing coh1C3 were capable of binding to UidA-dock1 (Figure 2, black bars), demonstrating the functionality of coh1C3 within recombinant chimeric scaffolds. Wild type UidA is tetrameric, and we considered, due to a 1:1 cohesin:dockerin binding ratio, that only one dockerin of each tetramer would bind a single cohesin of the same type on the chimeric scaffold. The inclusion of linkers at the C-terminus of coh1C3 seemed to have no significant effect on UidA-dock1 binding. A scaffold containing a single cohesin (CBD-coh1C3) was used as a reference point and showed that the addition of the coh2O2 cohesin to the synthetic scaffold reduced UidA-dock1 binding by ~two-fold (Figure 2). A strain displaying scaffold CBD alone or CBD-coh2O2 was also used as a negative control and failed to bind the UidA-dock1 reporter enzyme (Figure 2).

To test the functionality of the coh2O2 domain within the chimeric scaffolds, similar binding assays were carried out using UidA fused to a type 2 dockerin domain isolated from CipA (UidA-dock2). It has been previously demonstrated that OlpB is surface displayed on *C. thermocellum* and successfully binds the dock2 domain of CipA [15,47]. Surprisingly, the chimeric scaffolds containing the coh2O2 domain did not bind UidA-dock2 (Figure 2, white bars). All scaffolds lacking coh2O2 failed to bind UidA-dock2 as well. From these results, we hypothesized that either coh2O2 or dock2 were incapable of folding into their functional form when fused with CBD (alone or fused to coh1C3) or UidA, respectively. Since all tri-modular chimeric proteins did successfully bind UidA-dock1, we concluded that the lack of interaction of UidA-dock2 with the scaffolds was not due to lack of expression and secretion of the chimeric scaffold proteins, although fusion with coh2O2 did result in a two-fold reduction in successful scaffold secretion and anchoring. Background β-glucuronidase activity was slightly higher when using UidA-dock2 than when using UidA-dock1 (Figure 2). This residual β-glucuronidase activity can be attributed to a slightly higher non-specific adherence of UidA-dock2 to cells since binding of the fusion protein to the plasmid-free *L. lactis* strain showed similar levels of activity (data not shown). Based on these results, we sought to test if substitution of the type 2 cohesin domain in the chimeric scaffold would result in successful binding to UidA-dock2.

### CBD-coh1C3-coh2S1 chimeric scaffold binds UidA-dockerin fusion proteins

**Figure 3:**
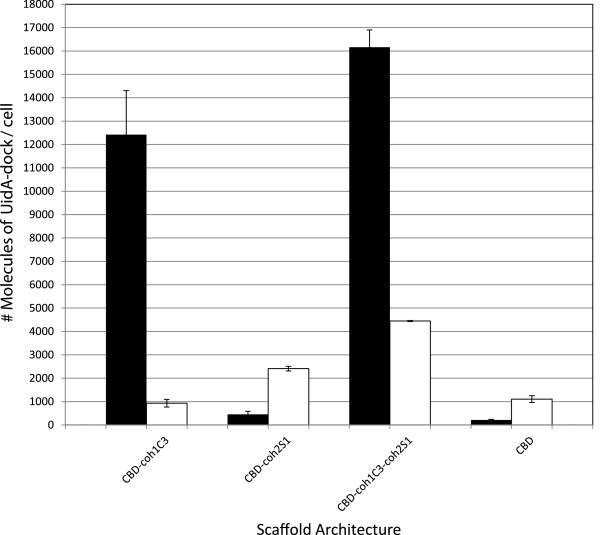
***In vivo *binding of UidA-dock1 and UidA-dock2 on *L. lactis *cells displaying coh1C3-coh2S1 chimeric scaffold proteins.** Cells displaying chimeric scaffold proteins were tested for their ability to bind UidA-dock1 or UidA-dock2. Quantification of enzymes was carried out using identical methods as described in the legend of figure 2. Bars represent the number of UidA-dock1 molecules (black bars) and UidA-dock2 molecules (white bars) successfully associated with the scaffolds.

Scaffolds engineered by replacing coh2O2 with the type 2 cohesin of SdbA (coh2S1) (Figure 1B) were capable of binding both UidA-dock1 and UidA-dock2 (Figure 3), establishing the functionality of coh2S1 domain incorporated into the chimeric scaffold protein. The coh2S1 domain was fused with either CBD alone or CBD-coh1C3. Substituting coh2O2 for coh2S1 therefore greatly improved UidA-dock2 binding to cells. Both OlpB and SdbA are anchor proteins that are responsible for binding CipA to the surface of *C. thermocellum*; however, these proteins have two striking differences. First, SdbA contains a single type 2 cohesin rather than four, and second, it contains a unique lysine-rich region at the C-terminus of the coh2S1 domain, which shows a high degree of homology to the streptococcal M proteins [14]. Both UidA-dockerin fusion proteins were able to bind the CBD-coh1C3-coh2S1 chimeric scaffold protein, demonstrating the functionality of both cohesin domains (Figure 3). Cells displaying CBD-coh1C3 or CBD-coh2S1 were only capable of binding UidA-dock1 and UidA-dock2 respectively, demonstrating the specificity of each cohesin-dockerin interaction. The cells that bound the greatest number of UidA-dockerin fusions were those displaying the larger tri-modular chimera CBD-coh1C3-coh2S1, which successfully bound 1.6 x 10^4^ molecules of UidA-dock1 / cell and 4.5 × 10^3^ molecules of UidA-dock2 / cell (Figure 3).

### CBD-coh1C3-coh2S1 chimeric scaffold binds LacZ-dockerin fusion proteins

**Figure 4:**
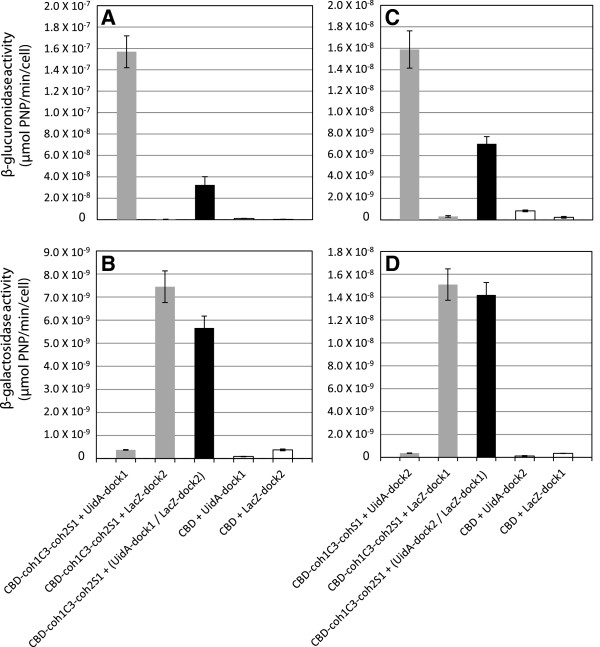
**β-glucuronidase and β-galactosidase activities of *L. lactis *cells with simultaneous targeting of UidA and LacZ to the scaffold proteins. **(**A** and **C**) β-glucuronidase and (**B** and **D**) β-galactosidase activity resulting from the simultaneous binding of (**A** and **B**) UidA-dock1 and LacZ-dock2 with a surface-displayed chimeric scaffold proteins or (**C** and **D**) resulting from the simultaneous binding of UidA-dock2 and LacZ-dock1 with a surface-displayed chimeric scaffold proteins. Enzyme activities are reported for a single enzyme bound to the scaffolds (grey bars), when both enzymes are bound (black bars), and with scaffolds lacking cohesin domains (white bars).

Having demonstrated the functionality of cell-displayed tri-modular synthetic scaffolds in binding a single enzyme, we sought to test the versatility of the scaffolds by binding a much larger enzyme. The *E. coli* β-galactosidase was fused to dock1 (LacZ-dock1) or dock2 (LacZ-dock2), and the resulting enzyme fusions were tested for their ability to bind the chimeric scaffolds. The LacZ-dockerin fusions were tested for their ability to bind cells displaying chimeric scaffold CBD-coh1C3-coh2S1 or CBD alone. Similar to UidA, LacZ is tetrameric, and we considered that due to a 1:1 cohesin:dockerin binding ratio only one dockerin would bind a single cohesin of the same type on the chimeric scaffold. LacZ-dock1 and LacZ-dock2 were both capable of binding the coh1C3 and coh2S1 sites on the chimeric scaffolds, respectively, and did not bind CBD (Figure 4B, D). This clearly indicated that much like the UidA-dockerin fusion proteins, the dockerin-containing LacZ was binding to its corresponding cohesin partner. LacZ lacking a dockerin domain did not bind to any of the strains described (data not shown). Having confirmed the functionality of the UidA and LacZ dockerin fusions, as well as their ability to bind to the chimeric scaffolds we sought to further probe the versatility of the scaffolds by binding two enzymes, simultaneously or sequentially.

### Simultaneous binding of UidA- and LacZ-dockerin fusions to chimeric protein scaffolds

The incubation of cells displaying the CBD-coh1C3-coh2S1 scaffold with an enzyme mixture consisting of equimolar amounts of both UidA-dock1 and LacZ-dock2 resulted in the successful assembly of a two-enzyme complex tethered to the surface of *L. lactis*. These results demonstrated that the architecture of the synthetic scaffold could accommodate both enzymes at the respective coh1C3 and coh2S1 sites. Comparisons in activity were made when each enzyme was targeted to the displayed scaffold independently, or when the two enzymes were bound simultaneously. Binding UidA-dock1 to the coh1C3 domain on the scaffold resulted in increased activity when compared with UidA-dock2 binding to the coh2S1 domain (Figure 4A, C), and this result was also observed when binding LacZ-dock1 and LacZ-dock2 to these same cohesin domains (Figure 4B, D). Since in our construct, coh1C3 is closer to the N-terminus of the protein, whereas coh2S1 is adjacent to the C-terminal cwa, it is possible that a greater protruding length of the scaffold exposing coh1C3 may have improved binding at this cohesin domain. The simultaneous binding of both UidA-dock1 and LacZ-dock2 resulted in a fivefold decrease in UidA activity compared to complexes containing UidA-dock1 alone (Figure 4A). In contrast, complexes containing both enzymes showed no significant (p>0.05, student’s T-test) decrease in LacZ activity when compared to complexes containing LacZ-dock2 alone (Figure 4B). To gain insight into the drop in enzyme activity observed for the two-enzyme complex, cells expressing the same scaffold were incubated with equimolar amounts of LacZ-dock1 and UidA-dock2, targeting the same enzymes to opposite cohesins. As observed previously, the simultaneous docking of both enzymes resulted in a decrease (two-fold) in UidA activity compared to the scaffolds to which only UidA-dock2 was bound (Figure 4C). Once more, no significant (p>0.05, student’s T-test) decrease in LacZ activity was observed for complexes containing both enzymes when compared with complexes containing LacZ-dock1 alone (Figure 4D).

### Sequential binding of UidA- and LacZ-dockerin fusions to chimeric protein scaffolds

**Figure 5:**
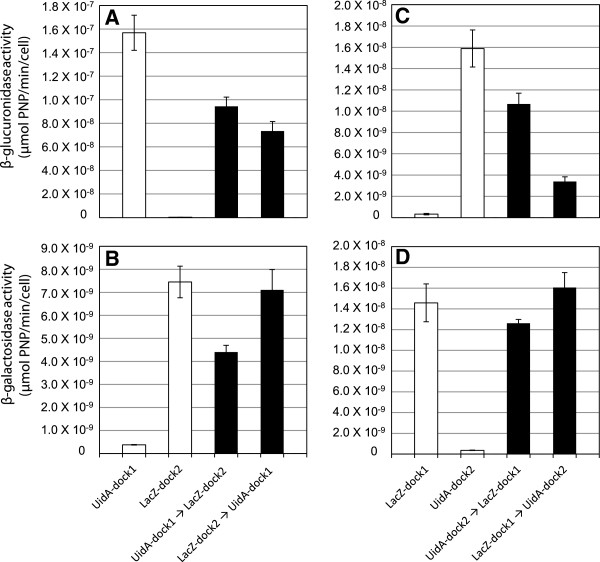
**β-glucuronidase and β-galactosidase activities of *L. lactis *cells with sequential targeting of UidA and LacZ to the scaffold proteins. **(**A** and **C**) β-glucuronidase and (**B** and **D**) β-galactosidase activities resulting from the sequential binding of reporter enzymes onto the chimeric scaffold CBD-coh1C3-coh2S1. (A and B) Sequential targeting of UidA-dock1 and LacZ-dock2 and (**C** and **D**) sequential targeting of UidA-dock2 and LacZ-dock1. Enzyme activities are reported for a single enzyme bound to the scaffolds (white bars) and when both enzymes are bound (black bars).

The order in which the chimeric scaffold was “loaded” with UidA and LacZ resulted in the assembly of two-enzyme complexes with different enzyme activities (Figure 5). When LacZ-dock2 was bound onto the scaffold CBD-coh1C3-coh2S1 prior to UidA-dock1, the result was a two-fold decrease in UidA activity when compared to similar complexes containing UidA-dock1 alone (Figure 5A). When this order of assembly was reversed, and UidA-dock1 was bound onto the scaffold chimera prior to LacZ-dock2, UidA activity was not significantly affected (p>0.05, student’s T-test) (Figure 5A). In the same experiment, β-galactosidase activity was also measured to determine the effect of the sequential incorporation of the two enzymes on LacZ activity. When UidA-dock1 was bound to the scaffold prior to LacZ-dock2, a similar result was observed where LacZ activity decreased approximately 1.8 fold compared to complexes containing LacZ-dock2 alone (Figure 5B). Contrarily, when LacZ-dock2 was bound to the scaffold prior to UidA-dock1, the resulting complex exhibited a similar level of LacZ activity when compared with complexes containing LacZ-dock2 alone (Figure 5B). When LacZ-dock1 was targeted to the scaffold prior to UidA-dock2, a 4.7-fold decrease in UidA activity was observed, compared to complexes containing UidA-dock2 alone (Figure 5C). However when the order was reversed and UidA-dock2 was incorporated prior to LacZ-dock1, much of the β-glucuronidase activity was regained, with an approximate 1.5 fold decrease in UidA activity compared with complexes containing UidA-dock2 alone (Figure 5C). Interestingly, the β-galactosidase activity of these complexes did not significantly (p>0.05, student’s T-test) change when the order of assembly was switched. When UidA-dock2 was incorporated into the complex prior to LacZ-dock1, the result was only a marginal decrease in LacZ activity when compared to complexes containing LacZ-dock1 alone (Figure 5D). In addition, when LacZ-dock1 was incorporated into the complex prior to UidA-dock2, LacZ activity was identical when compared with complexes containing LacZ-dock1 alone.

## Discussion

In a previous study, we reported on strains of *L. lactis* that successfully displayed type 1 cohesins on their surface, and demonstrated their ability to bind the β-glucuronidase-dockerin fusion protein UidA-dock1 [42]. In this study, chimeric scaffold proteins consisting of cohesins from CipA and OlpB or SdbA were successfully displayed on the surface of *L. lactis*, however only CipA-SdbA chimeric scaffolds were capable of binding both UidA-dock1 and UidA-dock2, suggesting that either improper folding or inaccessibility of coh2O2 may have prevented its association with UidA-dock2. Previous studies have demonstrated that scaffold proteins derived from bacteria that anchor their cellulosome to the cell surface such as *C. thermocellum, Ruminococcus flavifaciens,* and *Acetivibrio cellulolyticus*, contain long inter-cohesin linkers (50–550 residues) compared to cellulosomes from organisms which do not anchor their cellulosomes such as *Clostridium cellulolyticum* (10 residues) [12,48,49]. It has also been proposed that linkers joining cohesins within CipA may increase the protein’s conformational flexibility [50]. With the goal of improving coh2O2 accessibility for dockerin binding, scaffold-derived linkers were engineered in our synthetic scaffolds (Figure 1A), however no significant difference in enzyme binding at either cohesin was observed (Figure 2). Since the scaffolds were successfully displayed on the cell surface, we hypothesize that either improper folding of the scaffold protein may have resulted from unfavorable ionic interactions among amino acid residues, or that the coh2O2 domain remained buried within protein aggregates, ultimately inhibiting this cohesin’s ability to bind corresponding dockerin [51,52]. In addition, deletion of the HtrA housekeeping protease in our strain may account for the misfolded proteins remaining associated with the cell surface [53]. It has also been previously demonstrated that targeting recombinant fusion proteins to the cell wall of *L. lactis* can cause problems with secretion, anchoring, and/or folding [54].

Since the inclusion of linkers exterior to the coh2O2 domain did not result in binding of UidA-dock2 to the chimeric scaffolds, we replaced coh2O2 with coh2S1 and found that the resulting scaffold could bind UidA-dock1 and UidA-dock2 demonstrating that both cohesin domains were accessible and functional. SdbA differs from OlpB in that it contains one rather than four cohesins, as well as a lysine-rich region downstream of coh2S1 that shares a high degree of homology to a similar lysine-rich region of streptococcal M proteins located in our cwa_M6_, just upstream of the LPXTG sequence [14]. We postulate that incorporating coh2S1 adjacent to the anchor motif of streptococcal M6 protein may emulate some structural characteristics found in the native SdbA anchor protein of the *C. thermocellum* cellulosome, resulting in improved accessibility for UidA-dock2 binding. A total of four variant scaffolds (Figure 1B) containing both a type 1 and type 2 cohesin (CBD-coh1C3-coh2S1), only a type 1 cohesin (CBD-coh1C3), only a type 2 cohesin (CBD-coh2S1) or no cohesin (CBD alone) were tested for their ability to bind UidA-dock1 and/or UidA-dock2. Cells displaying CBD-coh1C3 were successful in binding UidA-dock1 but failed to bind UidA-dock2, while cells displaying CBD-coh2S1 successfully bound UidA-dock2 but failed to bind UidA-dock1, demonstrating the specificity of the interaction (Figure 3). Cells displaying the larger trimodular scaffold CBD-coh1C3-coh2S1 were capable of binding both UidA-dock1 and UidA-dock2. Interestingly, in the case of these larger scaffolds, the amounts of UidA-dock1 and UidA-dock2 molecules bound was greater when compared with cells displaying the smaller scaffolds CBD-coh1C3 and CBD-coh2S1, respectively (Figure 3). One possible explanation is that CBD-coh1C3-coh2S1 is secreted or displayed with increased efficiency, as in a previous study, we also demonstrated that increased scaffold protein size did not reduce the efficiency of scaffold display or functionality [42]. It also remains possible that better folding of each respective cohesin domain within CBD-coh1C3-coh2S1, when compared with the other constructs, may account for its ability to bind more UidA-dockerin fusion proteins.

Having determined the number of each UidA-dockerin fusion bound to displayed scaffold CBD-coh1C3-coh2S1, we analyzed their relative abundance within the assembled complexes, since protein ratios can ultimately have an effect on enzyme synergy and substrate-channeling [6,26]. Assuming a 1:1 cohesin to dockerin binding ratio, it would be expected that CBD-coh1C3-coh2S1 should bind equimolar amounts of UidA-dock1 and UidA-dock2. The resulting ratio deviated from this prediction, since the UidA-dock1 / UidA-dock2 ratio approached 4:1 (Figure 3). In a previous study, the assembly of chimeric scaffold-derived enzyme complexes on the surface of *Saccharomyces cerevisiae* also resulted in deviations from expected ratios of enzymes, as cellobiohydrolase CBHII associated with scaffolds at lower levels than other enzymes [11]. We therefore suggest that variability in the proper folding and/or accessibility of individual cohesin domains within a chimeric scaffold may affect binding of the enzymes to the scaffold.

To gain further insight into factors affecting protein binding to our synthetic scaffold proteins, we “docked” individual enzymes simultaneously or sequentially onto the chimeric CBD-coh1C3-coh2S1 protein. When simultaneously binding UidA-dock1 and LacZ-dock2 to the scaffold, an approximate five-fold decrease in UidA activity was observed compared to the binding of UidA-dock1 alone whereas no significant decrease in LacZ activity was observed in these assays (Figure 4). We hypothesize that the different effects on UidA and LacZ binding and/or activity may be due to either the location of the cohesin within the scaffold, to the size of each enzyme relative to the other, or differences in binding affinities between the two recombinant cohesin-dockerin interactions. Therefore, a similar binding assay was performed where the location of the cohesins on the scaffold protein was reversed. Similarly, UidA activity was two-fold lower when incorporated in the presence of LacZ-dock1, and once again, no significant change in LacZ activity was observed when incorporated in the presence of UidA-dock2 (Figure 4). Since LacZ is significantly larger than UidA (480 kDa vs 280 kDa), this suggests that enzyme size may result in steric factors inhibiting the binding of one enzyme partner, and that the relative location of each enzyme did not seem to play a role in the resulting activities when enzymes were incorporated simultaneously.

Sequential enzyme binding assays gave similar results as simultaneous binding assays where more than a two-fold decrease in UidA activity resulted when LacZ-dock2 was bound to the scaffold prior to UidA-dock1 addition. Contrarily, although LacZ activity decreased significantly when UidA-dock1 was bound to the scaffold protein prior to LacZ-dock2, reversing this order resulted in the same LacZ activity as when LacZ-dock2 alone was targeted to the scaffold (Figure 5). To verify if enzyme location also affected the overall resulting activity of the complex, the location of each enzyme partner was reversed. UidA activity decreased when LacZ-dock1 was incorporated prior to UidA-dock2, and this activity was only partially regained when the order of assembly was reversed (Figure 5C). LacZ activity was not affected by the order in which LacZ-dock1 and UidA-dock2 were bound into such complexes (Figure 5D). In addition, when UidA-dock1 was targeted to the coh1C3 cohesin (Figure 5A), the order in which LacZ was targeted to coh2S1 also had less of an effect on resulting UidA activity compared to when UidA-dock2 was targeted to coh2S1 (Figure 5C). From these results, it appears that when a fusion enzyme is targeted to the outermost position on the scaffold, distal to the cell surface, its binding to the scaffold may be less affected by enzyme partners, compared to when it is targeted to the innermost position, proximal to the cell surface.

## Conclusions

We describe the first successful display of engineered chimeric scaffolds containing type 1 and type 2 cohesins on the surface of *L. lactis,* and the ability for the scaffolds to support the assembly of multi-enzyme complexes. Traditional modes of enzyme display in lactic acid bacteria were generally limited to fusing a single enzyme with an appropriate anchor [38-41,55-62]. In this study, we expand this capacity to two enzymes with the simultaneous or sequential incorporation of the two enzymes resulting in differences in the enzymatic profile of the assembled complexes. These results suggest that the size and location of each enzyme within each complex should be carefully taken into consideration when further developing this system of enzyme display. We envision that this system could have potentially broad implications in a number of complex bioconversion processes including the degradation of complex polymers, and the synthesis of complex molecules.

## Methods

### Bacterial strains and plasmids

The bacterial strains and plasmids used in this study are listed in Table 1. *E. coli* strains were grown in Luria-Bertani medium at 37°C with shaking (220 rpm). *Lactococcus lactis htra* NZ9000 was grown in M17 medium [63] supplemented with 1% (w/v) glucose (GM17) at 30°C without agitation*.* To make competent cells, *L. lactis* was grown in GM17 medium supplemented with 25% (w/v) sucrose and 2% (w/v) glycine and cells were transformed as previously described [64]. *C. thermocellum* was grown in ATCC1191 medium at 55°C with 0.2% (w/v) cellobiose as a carbon source. Genomic DNA was isolated from *C. thermocellum* as previously described [65]. Where appropriate, antibiotics were added as follows: for *E. coli*, ampicillin (100 μg/mL), chloramphenicol (10 μg/mL) and kanamycin (30 μg/mL); for *L. lactis,* erythromycin (5 μg/mL) and chloramphenicol (10 μg/mL). General molecular biology techniques for *E. coli* were performed as previously described [66].

**Table 1:** Strains and plasmids used in this study

**Strain**	**Genotype / Description**	**Source**
*L. lactis htrA* NZ9000	Mutant MG1363 derivative (*nisRK* genes on the chromosome) lacking *htrA*	[68]
*E. coli* TG1	*supE thi-1* Δ(*lac-proAB*) Δ(*mcrB-hsdSM*)*5* (rK– mK–) [F′ *traD36 proAB lacI*q*Z*Δ*M15*]	ATCC
*E. coli* BL21 (DE3)	*F*^*–*^*ompT gal dcm lon hsdS*_*B*_*(r*_*B*_^*-*^*m*_*B*_^*-*^*) λ(DE3 [lacI lacUV5-T7 gene 1 ind1 sam7 nin5])*	Novagen
**Plasmid**		
pET28(b)	Km^r^	Novagen
pAW528	Cm^r^, Amp^r^; pBS::pIL252::*t*_*trpA*_::*P*_*nisA*_::*rbs*_*nisA*_::*sp*_Usp45_*- CBD-coh1C3-cwa*_M6_*-tlt2*	[42]
pAW531	Cm^r^, Amp^r^; pBS::pIL252::*t*_*trpA*_::*P*_*nisA*_::*rbs*_*nisA*_::*sp*_Usp45_*-CBD-cwa*_M6_*-tlt2*	[42]
pAW549	Cm^r^, Amp^r^; pBS::pIL252::*t*_*trpA*_::*P*_*nisA*_::*rbs*_*nisA*_::*sp*_Usp45_*-CBD-coh2O2-cwa*_M6_*-tlt2*	This Work
pAW564	Cm^r^, Amp^r^; pBS::pIL252::*t*_*trpA*_::*P*_*nisA*_::*rbs*_*nisA*_::*sp*_Usp45_*-CBD-Lk-coh2O2-cwa*_M6_*-tlt2*	This Work
pAW596	Cm^r^, Amp^r^; pBS::pIL252::*t*_*trpA*_::*P*_*nisA*_::*rbs*_*nisA*_::*sp*_Usp45_*-CBD-coh2O2-Lk-cwa*_M6_*-tlt2*	This Work
pAW594	Cm^r^, Amp^r^; pBS::pIL252::*t*_*trpA*_::*P*_*nisA*_::*rbs*_*nisA*_::*sp*_Usp45_*-CBD-Lk-coh2O2-Lk-cwa*_M6_*-tlt2*	This Work
pAW546	Cm^r^, Amp^r^; pBS::pIL252::*t*_*trpA*_::*P*_*nisA*_::*rbs*_*nisA*_::*sp*_Usp45_*-CBD-coh1C3-coh2O2-cwa*_M6_*-tlt2*	This Work
pAW561	Cm^r^, Amp^r^; pBS::pIL252::*t*_*trpA*_::*P*_*nisA*_::*rbs*_*nisA*_::*sp*_Usp45_*-CBD-coh1C3-Lk-coh2O2-cwa*_M6_*-tlt2*	This Work
pAW595	Cm^r^, Amp^r^; pBS::pIL252::*t*_*trpA*_::*P*_*nisA*_::*rbs*_*nisA*_::*sp*_Usp45_*-CBD-coh1C3-coh2O2-Lk-cwa*_M6_*-tlt2*	This Work
pAW592	Cm^r^, Amp^r^; pBS::pIL252::*t*_*trpA*_::*P*_*nisA*_::*rbs*_*nisA*_::*sp*_Usp45_*-CBD-coh1C3-Lk-coh2O2-Lk-cwa*_M6_*-tlt2*	This Work
pAW579	Cm^r^, Amp^r^; pBS::pIL252::*t*_*trpA*_::*P*_*nisA*_::*rbs*_*nisA*_::*sp*_Usp45_*-CBD-coh2S1-cwa*_M6_*-tlt2*	This Work
pAW576	Cm^r^, Amp^r^; pBS::pIL252::*t*_*trpA*_::*P*_*nisA*_::*rbs*_*nisA*_::*sp*_Usp45_*-CBD-coh1C3-coh2S1-cwa*_M6_*-tlt2*	This Work
pETdock1	Kn^r^; pET28(b)::with cloned *dock1* from *celS*	[42]
pETdock2	Kn^r^; pET28(b)::with cloned *dock2* from *cipA*	This Work
pETUdock1	Kn^r^; pET28(b)::*PT7*::*6xHis-uidA-dock1*	[42]
pETUdock2	Kn^r^; pET28(b)::*PT7*::*6xHis-uidA-dock2*	This Work
pETU	Kn^r^; pET28(b)::*PT7*::*6xHis-uidA*	[42]
pETLdock1	Kn^r^; pET28(b)::*PT7*::*6xHis-lacZ-dock1*	This Work
pETLdock2	Kn^r^; pET28(b)::*PT7*::*6xHis-lacZ-dock2*	This Work
pETL	Kn^r^; pET28(b)::*PT7*::*6xHis-lacZ*	This Work

The pAW500 series of vectors are designed for the cell-wall targeting of various scaffold protein permutations consisting of cohesins from CipA and OlpB or SdbA. *coh1C3*, type 1 cohesin of CipA; *coh2O2*, type 2 cohesin of OlpB; *coh2S1*, type 2 cohesin of SdbA; *PT7*, inducible T7 promoter; *P*_*nisA*_, inducible *nisA* promoter; *rbs*_*usp45*_, Usp45 ribosome-binding site; *rbs*_*nisA*_, *nisA* ribosome-binding site; *sp*_Usp45_, signal sequence of Usp45; *cwa*_M6_, anchor motif of M6 protein; *tlt2*, transcriptional terminator of *rrnB* operon; *t*_*trpA*_, transcriptional terminator of *trpA*; *Lk, olpB* linker region.

### Assembly of chimeric scaffolds expression cassettes

The *E. coliL. lactis* shuttle vectors pAW528 and pAW531 both contain gene expression cassettes for the secretion and surface display of the scaffold proteins (Table 1) [42]. Scaffolds are expressed as fusions with the N-terminal signal peptide from the lactococcal Usp45 (Genbank Accession no. AAA25230.1) secreted protein (sp_Usp45_) [43] and with the C-terminal anchor motif of streptococcal M6 protein (Genbank accession no. AAA26920.1) cwa_M6_[60]. Expression of the cassettes is under the control of the *nisA* nisin-inducible promoter (*P*_*nisA*_) and ribosome-binding site (*rbs*_*nisA*_) from *L. lactis*[44,45]. For the construction of cassettes encoding chimeric protein scaffolds, PCR was performed on *C. thermocellum* genomic DNA to amplify regions encoding fragments of the cellulosomal proteins CipA (GenBank accession no. Q06851), OlpB (GenBank accession no. CAA47841.1) and SdbA (GenBank accession no. AAB07763.1). DNA encoding the second cohesin of OlpB (coh2O2) was amplified using primers a and b (Table 2). In order to incorporate the protein linker sequence at the N-terminal end of the coh2O2 cohesin, link-coh2O2 was amplified using primers c and b. For incorporation of the C-terminal linker into the recombinant scaffold, coh2O2-link was amplified using primers *a* and *d*. To engineer a scaffold with the coh2O2 cohesin flanked by two linkers, the *link-coh2O2-link* fragment was amplified using primers *c* and *d*. PCR products were purified using a PCR purification kit (Qiagen), digested with *Not*I and *EcoR*I, and ligated into similarly cut pAW528 and pAW531, yielding vectors pAW549 (CBD-coh2O2), pAW546 (CBD-coh1C3-coh2O2), pAW564 (CBD-Link-coh2O2), pAW561 (CBD-coh1C3-Link-coh2O2), pAW596 (CBD-coh2O2-Link), pAW591 (CBD-coh1C3-coh2O2-Link), pAW594 (CBD-Link-coh2O2-Link) and pAW592 (CBD-coh1C3-Link-coh2O2-Link) (Table 1).

**Table 2:** Primers used in this study. Restriction enzyme cut sites are in bold

**Primer**	**Sequence (5’ – 3’)**
*a*	TCGA**GCGGCCGC**GCTGGAACTGGATAAGAC
*b*	TCGA**GATATC**TTAGGCTGTACTACGCTATAC
*c*	TCGA**GCGGCCGC**GCTTATAGTTGTAGAGGC
*d*	ATGC**GATATC**GTCGACTTTATTACATAGGAATCTGGAAG
*e*	TCGA**GCGGCCGC**GGATAAAGCCTCGAGCATTG
*f*	TCGA**GATATC**TTATCCGGCTGTATTACCTC
*g*	ATGC**GAATTC**GGAGACATAGTGAAAGACAATTC
*h*	ATGC**GCGGCCGC**TTTACTGTGCGTCGTAATCAC
*i*	ATGC**GCTAGC**ATGACCATGATTACGG
*J*	GCAT**CAATTG**TTTTTGACACCAGACC

Type 2 cohesin coh2S1 of anchor protein SdbA was PCR-amplified using primers *e* and *f*, purified using a PCR purification kit (Qiagen), digested with *Not*I and *Eco*RV, and ligated to similarly cut pAW528 and pAW531, yielding pAW576 (CBD-coh1C3-coh2S1) and pAW579 (CBD-coh2S1), respectively (Table 1).

### Assembly of dockerin-fused UidA and LacZ expression cassettes

*E. coli* β-glucuronidase (UidA, GenBank accession no. ZP_03034971.1) was previously engineered to contain a C-terminal dock1 domain for binding of the enzyme to type 1 cohesins [42]. In this study, UidA was fused with a dock2 domain from CipA for binding to type 2 cohesins, as well as an N-terminal 6 x His-tag for protein purification. For assembly of the *hisX6-uidA-dock2* cassette, the dock2 sequence of the *cipA* gene was amplified from *C. thermocellum* genomic DNA using primers *g* and *h* (Table 2). The PCR product was digested with *Eco*RI-*Not*I and ligated to similarly-digested pET28(b), yielding pETdock2. To create the UidA-dock2 fusion, pETUdock1 was digested with *Nhe*I-*Eco*RI to isolate the *uidA* gene, which was gel-purified, and ligated to similarly cut pETdock2, yielding pETUdock2. In order to create LacZ-dockerin fusion proteins, DNA encoding the *E. coli* β-galactosidase LacZ (GenBank accession no. EGT70540.1) was PCR amplified from genomic DNA of *E. coli* MG1655 using primers *i* and *j*. The resulting PCR product was digested with *Nhe*I-*Mfe*I and ligated into *Nhe*I-*Eco*RI-digested pETU, pETUdock1 and pETUdock2, yielding pETL, pETLdock1 and pETLdock2, respectively (Table 1). All pET vectors described above express cassettes encoding enzymes and enzyme-dockerin fusions with an N-terminal 6XHis tag for purification.

### Expression and purification of dockerin-fused UidA and LacZ

All His-tagged enzymes were expressed in *E. coli* BL21(DE3) as previously described [42]. The UidA and LacZ-containing elution fractions were identified by the appearance of a yellow color in a liquid β-glucuronidase and β-galactosidase assay, respectively. Liquid β-glucuronidase assay conditions are previously described [42]. For liquid β-galactosidase assay, 50 μL of each elution fraction were added to 450 μL of Z buffer containing 100 mM phosphate buffer pH7, 5 mM KCl, 1 mM MgSO_4_, 0.28% (v/v) β-mercaptoethanol. Samples were heated for 1 min, after which *p*-nitrophenyl-β-D-glucuronide was added to a final concentration of 4 mg/mL [67]. The purity of the elution fractions exhibiting UidA and LacZ activity was assessed by SDS-PAGE (12%, w/v). Proteins were stained using Coomasie Blue Reagent (BioRad) and fractions containing the highest purity of enzyme were pooled. The specific activities of UidA-dock1 and UidA-dock2 were determined by colorimetric assays in a thermostated UV–vis spectrophotometer (Cary 50 WinUv) at 405 nm, using a 1 cm (L) cuvette, and the molar extinction coefficient of *p*-nitrophenyl (PNP) being 18 000 M^-1^ cm^-1^. A Bradford protein assay kit (Pierce) and BSA as a standard were used in order to quantify net protein amounts, and specific activities were used to evaluate the amount of enzyme bound to cells in the *in vivo* binding assay described below. For simultaneous or sequential binding assays, overall enzymatic activities/cell were calculated by measuring colorimetric changes using *p-*nitrophenyl-β-D-glucuronide as substrate and 405 nm wavelength for UidA activity, and *O*-nitrophenyl-β-galactoside as substrate and 420 nm wavelength for LacZ activity.

### Quantitation of UidA-dockerin binding to *L. Lactis*-expressed scaffold proteins

*L. lactis htrA* NZ9000 was transformed with the expression plasmids encoding permutations of chimeric scaffolds (Figure 1). The strain is deficient in the HtrA extracellular protease and contains chromosomal copies of the *nisR* and *nisK* genes, which participate in the regulation of expression cassettes under control of the *nisA* promoter [68]. *L. lactis* cells harboring the plasmids were grown overnight in GM17 medium and diluted 1/50 in 5 mL of fresh media and grown for an additional 4 hrs (OD_600_ ≈0.3) after which cells were induced with 10 ng nisin/mL for scaffold expression [42]. After 20 hrs growth, 1 mL of cells were washed in phosphate buffer (50 mM, pH 6.0) containing 300 mM NaCl and suspended in 100 μL of purified UidA-dock1 or UidA-dock2 at a concentration of 100 μg/mL. Binding assay conditions and enzyme quantification methods used to determine the amount of enzyme associated with *L. lactis* cells are previously described [42]. The specific activities of UidA-dock1 and UidA-dock2 were determined to be 25 μmol PNP mg^-1^ min^-1^ and 13 μmol PNP mg^-1^ min^-1^, respectively. Using the calculated molecular weights of UidA-dock1 and UidA-dock2 and the known amount of cells present in each sample, the average number of enzyme units bound per cell was estimated. Experiments were performed in triplicate using true biological replicates (independent colonies and cultures).

### Simultaneous or sequential binding of UidA- and LacZ-dockerin to cells displaying chimeric protein scaffolds

Enzyme combinations consisting of equimolar amounts of UidA-dock1 and LacZ-dock2 or UidA-dock2 and LacZ-dock1 were mixed to a final enzyme concentration of 100 μg/mL. Cells were incubated in 100 μL of the enzyme mixture, washed 6 times in phosphate buffer (50 mM, pH 6) containing 300 mM NaCl, re-suspended in 100 μL of the same buffer, and analyzed using both the β-glucuronidase assay [42] and β-galactosidase assay. For sequential binding assays, cells were incubated with a first test enzyme at a concentration of 100 μg/mL, or no enzyme. After 5 hours of incubation at 4°C, cells were harvested by centrifugation and suspended in 100 μL phosphate buffer (50 mM, pH 6) containing 300 mM NaCl and an equimolar amount of the second enzyme or no enzyme. After an additional 5 hours of incubation, cells were harvested, washed 6 times in phosphate buffer (50 mM, pH 6) containing 300 mM NaCl, suspended in 100 μL of the same buffer, and tested for both β-glucuronidase and β-galactosidase activity.

### Ethical approval and consent

No human or animal subjects were used in this study. All experimental procedures have been carried out in compliance with the ethical standards of Concordia University's Office of Research.

## Competing interests

The authors declare they have no competing interests.

## Authors’ contributions

VM defined the strategy described and supervised the project. AW designed and carried out all experiments. AW drafted the initial manuscript and both AW and VM edited final manuscript. VM supervised the entire PhD project of AW. All authors read and approved the final manuscript.
